# Epigenetic regulation of Delta-Like1 controls Notch1 activation in gastric cancer

**DOI:** 10.18632/oncotarget.414

**Published:** 2011-12-31

**Authors:** Giulia Piazzi, Lucia Fini, Michael Selgrad, Melissa Garcia, Yahya Daoud, Thomas Wex, Peter Malfertheiner, Antonio Gasbarrini, Marco Romano, Richard L. Meyer, Robert M. Genta, James G. Fox, C. Richard Boland, Franco Bazzoli, Luigi Ricciardiello

**Affiliations:** ^1^ Department of Clinical Medicine, University of Bologna, Bologna, Italy; ^2^ Center for Applied Biomedical Research (CRBA), S.Orsola-Malpighi Hospital, University of Bologna, Bologna, Italy; ^3^ Department of Internal Medicine, Baylor Research Institute and Sammons Cancer Center, Baylor University Medical Center, Dallas, USA; ^4^ Department of Gastroenterology, IRCCS, Istituto Clinico Humanitas, Rozzano, Milan, Italy; ^5^ Department of Gastroenterology, Hepatology and Infectious Diseases, Otto-von-Guericke University Magdeburg, Magdeburg, Germany; ^6^ Institute for Health Care Research and Improvement Baylor Health Care System, Baylor University Medical Center, Dallas, USA; ^7^ Internal Medicine and Gastroenterology, Catholic University, Rome, Italy; ^8^ Department of Internal Medicine-Gastroenterology, Second University of Naples, Italy; ^9^ Department of Pathology, Baylor University Medical Center, Dallas, USA; ^10^ Department of Pathology, VA Medical Center, Dallas, USA; ^11^ Division of Comparative Medicine and Department of Biological Engineering, Massachusetts Institute of Technology, Cambridge, USA

**Keywords:** Gastric cancer, Methylation, Notch, Delta like-1

## Abstract

The Notch signaling pathway drives proliferation, differentiation, apoptosis, cell fate, and maintenance of stem cells in several tissues. Aberrant activation of Notch signaling has been described in several tumours and in gastric cancer (GC), activated Notch1 has been associated with de-differentiation of lineage-committed stomach cells into stem progenitors and GC progression. However, the specific role of the Notch1 ligand (DLL1) in GC has not yet been elucidated. To assess the role of DLL1 in GC cancer, the expression of Notch1 and its ligands DLL1 and Jagged1, was analyzed in 8 gastric cancer cell lines (KATOIII, SNU601, SNU719, AGS, SNU16, MKN1, MKN45, TMK1). DLL1 expression was absent in KATOIII, SNU601, SNU719 and AGS. The lack of DLL1 expression in these cells was associated with promoter hypermethylation and 5-aza-2’deoxycitidine caused up-regulation of DLL1. The increase in DLL1 expression was associated with activation of Notch1 signalling, with an increase in cleaved Notch1 intracellular domain (NICD) and Hes1, and down-regulation in Hath1. Concordantly, Notch1 signalling was activated with the overexpression of DLL1. Moreover, Notch1 signalling together with DLL1 methylation were evaluated in samples from 52 GC patients and 21 healthy control as well as in INS-GAS mice infected with *H. pylori* and randomly treated with eradication therapy. In GC patients, we found a correlation between DLL1 and Hes1 expression, while DLL1 methylation and Hath1 expression were associated with the diffuse and mixed type of gastric cancer. Finally, none of the samples from INS-GAS mice infected with *H. pylori,* a model of intestinal-type gastric tumorigenesis, showed promoter methylation of DLL1. This study shows that Notch1 activity in gastric cancer is controlled by the epigenetic silencing of the ligand DLL1, and that Notch1 inhibition is associated with the diffuse type of gastric cancer.

## INTRODUCTION

Although there is an overall worldwide decline in incidence, gastric cancer (GC) is still the fourth more common cancer and the second leading cause of cancer-related deaths [1]. It is known that the risk factors for this disease include diet, *Helicobacter (H.) pylori* infection and genetic alterations [2-3]. To date, the mechanisms controlling GC aggressiveness are not fully elucidated.

Notch signaling is a key pathway in self-renewal of stem cells, cell fate determination and terminal differentiation of proliferating cells [4]. The mammalian genome encodes for four Notch receptors (Notch 1-4) and five Notch ligands (Delta-Like1, Delta-Like3, Delta-Like4, Jagged1 and Jagged2). After ligand binding, the Notch extracellular domain is cleaved by the ADAM proteases and then subjected to further cleavage by the γ-secretase complex with the release of the Notch intracellular domaine (NICD). After translocating into the nucleus, NICD acts as a transcription factor for its downstream targets [5-8]. The best-characterized Notch effectors are the bHLH protein Hairy/Enhancer of Split (HES), which suppress the expression of downstream genes such as Neurogenin3 and Hath1. Importantly, these genes play a critical role in cell lineage commitment and drive cell fates and differentiation in several tissues [8-9]. In particular, in the gastrointestinal (GI) tract, Notch signaling drives the fate of immature progenitors toward absorptive or secretory lineage. In the intestine, Notch1 controls the differentiation towards enterocytes, and its inhibition converts colonic proliferative crypt cells into post-mitotic goblet cells [8]. Moreover, Hes1^−/−^ mutant mice develop intestinal abnormalities with a relative increase in mucus secreting and enteroendocrine cells at the expense of absorptive enterocytes, whereas Math1-deficient mice display a ‘reciprocal phenotype’ [10-11]. In the stomach, Notch activity is involved in the inhibition of chief cell differentiation and Hath1 over-expression enhances MUC5AC expression in the mucous neck cells of the fundic glands [10, 12]. Notch signaling occurs in the mouse stomach during development and is restricted to the isthmus in adult glands. Recently, Notch activation in lineage committed stomach epithelial cells has been shown to induce de-differentiation into stem cells, enhances proliferation and adenomas whereas Notch activation in the antral portion of the stomach does not affect proliferation [13].

Among the entire panel of the Notch ligands, Delta-Like1 (DLL1) has been demonstrated to be frequently involved in the assignment of the cell lineage fate. In zebrafish intestines, the inhibition of Delta-Notch signaling enhances the secretory differentiation [14]. Interestingly, inactivation of DLL1 in mice intestinal tissue mimics the inactivation of Notch1 as it results in an increase number of goblet cells, indicating that DLL1 is critical in the control of Notch1 in this system [15]. However, the interaction between DLL1 and Notch1 is responsible for regulating glandular differentiation in chicken stomach development [16]. Finally, mice deficient in DLL1 have an accelerated differentiation of pancreatic endocrine cells at the expenses of the exocrine component [17].

In this study we assessed the role of DLL1-Notch1 signaling in gastric carcinogenesis, given that an imbalance of self-renewal homeostasis is an essential requirement for tumorigenesis and deregulated expression of Notch signaling components frequently occurs in tumors [7, 18]. For this purpose, we evaluated the Notch1 cascade in gastric cancer cell lines and we found a specific correlation between DLL1 expression and promoter hypermethylation. The relationship between DLL1 expression and Notch1 activation was demonstrated *in vitro* by pharmacological studies and by utilizing transgenic models. Moreover, our *in vivo* data showed that the epigenetic silencing of DLL1 with repression of the Notch1 cascade was associated with diffuse gastric carcinogenesis. These results were further confirmed by the INS-GAS murine model of gastric intestinal carcinogenesis, in which DLL1 was expressed and promoter methylation was absent.

## MATERIALS AND METHODS

### Cell lines and treatments

Gastric cancer (GC) cell lines AGS, KATOIII, SNU16, SNU601, TMK1 and MKN45 were a kind gift from Dr. Antonia R. Sepulveda; MKN1 was kindly provided by Dr. Richard Hamelin; SNU719 was kindly given by Dr. Dong K. Chang. STR profiling was performed for cell lines authentication.

Cells were cultured in RPMI-1640 Medium (Invitrogen, Carlsbad, CA) supplemented with 10% of fetal bovine serum (Invitrogen), 100 U/ml penicillin, 100 μg/ml streptomycin and 2mM glutamine (Invitrogen). Cells were maintained at 37°C in a 5% CO_2_ incubator.

5-aza-2’deoxycitidine was purchased by Sigma-Aldrich (St.Louis, MO) and the treatment was performed on AGS and SNU719 cell lines at 10 μM for 5 days.

The full DLL1 coding sequence was cloned into the pCMV-Tag1 expression vector (Stratagene, La Jolla, CA), using BamHI and HindIII restriction enzymes (NEB, Ipswich, MA). Transient overexpression of DLL1 was performed using Lipofectamine 2000 (Invitrogen), following the protocol suggested by the manufacturer.

### Clinical gastric tissue samples

Formalin fixed, paraffin embedded tissues from 52 gastric cancer patients were obtained from the Department of Pathology at Baylor University Medical Center (Dallas, TX, USA), and from the Department of Gastroenterology, Hepatology and Infectious Disease, Otto-von-Guericke at University of Magdeburg (Magdeburg, Germany). Forty-three fresh gastric tumor samples, their matched normal counterparts and 21 biopsies from healthy subjects were obtained during endoscopic procedures at the Department of Gastroenterology, Hepatology and Infectious Disease, Otto-von-Guericke at University Magdeburg. Informed consent was obtained prior to procedures. GC histopathological characterization and staging of the patients were also collected. Institutional Review Board approval was granted for this study from Baylor University Medical Center and Otto-von-Guericke Department at University Magdeburg.

### RNA extraction, RT-PCR and quantitative PCR

RNA extraction from GC cell lines and tissues was performed with TRIzol (Invitrogen) and 2 μg of RNA were retrotranscribed using random hexamers and MMLV reverse transcriptase (Invitrogen). RT-PCR was performed using the HotstarTaq Master Mix Kit (Qiagen,Valencia, CA). Primers sequences are reported in Table 1. PCR products were separated on a 2% agarose gel, stained with 0.5 μg/ml ethidium bromide and the image was acquired under UV illumination (Gel Logic Imaging System, Rochester, NY).

Relative quantification of HES1 and HATH1 was performed by quantitative PCR (qPCR) using TaqMan Gene Expression Master Mix (Applied Biosystems, Foster City, CA) and the TaqMan Gene Expression Assay for HES1 (Hs 00172878-m1) and HATH1 (Hs 00245453-s1). GAPDH was used as the reference gene. The relative quantification of gene expression was performed with the comparative CT method (2^−ΔΔCt^), using the correspondent *wild-type* cell line or a pool of healthy subjects as a calibrator. Each evaluation was performed in triplicate in three independent experiments.

**Table 1:** Primers

Gene	Primer Forward (5’-3’)	Primer Reverse (5’-3’)	Size (bp)
DLL1	TATCCGCTATCCAGGCTGTC	GGTGGGCAGGTACAGGAGTA	297
Notch 1	CAGGCAATCCGAGGACTATG	CAGGCGTGTTGTTCTCACAG	429
Jagged 1	TCGCTGTATCTGTCCACCTG	AGTCACTGGCACGGTTGTAG	227
Beta-Actin	TCACACTGGCATCGTGATGGACTC	TCCTGCTTGCTGATCCACATCTGC	642
DLL1 Bisulfite	GGTTTTTAAAGAAAGAAGTTTTGGG	CCCAAAACTCCAAACCTACAC	501
DLL1 MSP M Reg1	ATATTCGTCGTCGTCGATC	CCGAACCGATTAAAAAACC	100
DLL1 MSP U Reg1	GTATATTTGTTGTTGTTGATT	TCCCAAACCAATTAAAAAACC	100
DLL1 MSP M Reg2	AAGGGCGTTTTTTTGTTTAC	ATACTACTTCGCTCCACGC	114
DLL1 MSP U Reg2	GGTAAGGGTGTTTTTTTGTTTAT	ATACTACTTCACTCCACACACA	114
DLL1 Mouse	CTGTGACAAACCAGGGGAGT	GACAACCTGGGTATCGGATG	110
GAPD Mouse	TCGGTGTGAACGGATTTGGC	GGTCGTTGATGGCAACAATC	90
DLL1 Bisulfite Reg1 Mouse	TGGTATTGGTTGAATTTTTGAG	CCCAAATATTCAACTTAATTCCC	273
DLL1 Bisulfite Reg2 Mouse	TTTTGGGTTTTTGAAGAAGAAA	CCCAACAACCCCTTCTTATTA	408
DLL1 MSP Reg1 M Mouse	GTAGCGGTTGTCGAGTGAC	ACCGATAAAACGATAATCCG	112
DLL1 MSP Reg1 U Mouse	GGTAGTGGTTGTTGAGTGAT	CACCAATAAAACAATAATCCA	112
DLL1 MSP Reg2 M Mouse	TAAGTGATTTCGGTAGCGAC	ACTAAAACGCAAAAACCGA	96
DLL1 MSP Reg2 U Mouse	TTTTAAGTGATTTTGGTAGTGAT	TACTAAAACACAAAAACCAAAC	96

### Protein extraction and western blotting

Total protein extraction from cell lines was performed using RIPA Buffer (Santa Cruz Biotechnology, Santa Cruz, CA) supplemented with protease and phophatase inhibitors (Roche, NJ, USA). Forty micrograms of proteins were separated on a 10% SDS-PAGE, transferred onto PVDF membrane (GE Healthcare, NJ, USA) and probed overnight with the following primary antibodies: rabbit anti cleaved Notch1 1:1000 (Cell Signaling Technology, Boston, MA), rabbit anti Delta 1:100 (H-265, Santa Cruz Biotechnology) and mouse anti-β actin 1:1000 (Sigma-Aldrich). After washing with TBS-T, the membranes were incubated with the anti-rabbit or anti-mouse IgG–horseradish peroxidase (GE Healthcare) secondary antibody at the concentration of 1:5000 for 45 minutes at room temperature. Proteins were visualized using the ECL Plus Chemiluminescence system and the membranes were scanned with a STORM 840 Phosphoimager (GE Healthcare). The housekeeping protein β-actin was used to normalize protein expression levels.

### MSP and bisulfite sequencing of DLL1 promoter

DNA was extracted using the QIAamp DNA Mini kit extraction kit according to the manufacturer’s protocol (Qiagen). The methylation status of the DLL1 promoter in GC cell lines and tissues was determined by bisulfite sequencing and methylation specific-PCR (MSP) after treating 1 μg of DNA with sodium bisulfite with the Epitect Bisulfite Kit (Qiagen), following the manufacturer’s protocol. Modified DNA was used as a template for PCR reactions. For the Bisulfite Sequencing, primers amplifying a sequence located between -158 and +343 from the transcriptional start codon and containing 56 CpGs were selected. PCR amplification was performed for 14 cycles with annealing temperature of 56.6 °C touching down of 0.5°C per cycle for 30 seconds and an additional 19 cycles with an annealing temperature of 49.6°C for 30 seconds. Each cycle started with a denaturation at 95°C and ended with an extension at 72°C, each for 30 seconds. Two microliters of PCR products were ligated into the TOPO vector (Invitrogen, Carlsbad, CA) and, after transformation, at least 8 clones per sample were sequenced in both directions with M13 primers. MSP was performed in two different regions of the promoter, spanning respectively between -532 and -432 (Reg1) and – 112 and the ATG start codon (Reg2). Primers sequences are listed in Table 1.

### Methylation and expression analysis of DLL1 in INS-GAS mice

Twelve INS-GAS mice were previously infected with *H. pylori* and treated at prescribed intervals with antibiotic eradication therapy administered at 8 (n=3), 12 (n=3) or 22 (n=3) weeks post *H. pylori* infection (WPI) or not treated (n=3) [19]. DNA was extracted from gastric tissues of the 12 *H. pylori* infected mice and 3 control mice (not infected and not treated) and modified with bisulfite treatment as above. DLL1 promoter hypermethylation was analyzed by Bisulfite Sequencing in two representative areas of the promoter (Reg1: from -617 to -344) and first exon (Reg2: from -65 to +220). MSP was performed in two different regions of the promoter, spanning respectively between -538 to -426 (Reg1) and +119 to +215 (Reg2). RNA was extracted from gastric tissues and retrotranscribed as above. DLL1 expression was evaluated by RT-PCR. Primers sequences are listed in Table 1.

### Statistical analysis

Unpaired T-test was used to evaluate the mean differences among two groups for the continuous variables. Median Test was used to evaluate the median differences among two groups for the continuous variables. Analysis of variance (ANOVA) was used to evaluate the mean differences among three groups for the continuous variables. LSD all Pairwise Comparison Test was applied to compare groups of continuous variables. Fisher Exact test was applied to analyze categorical variables. Correlation analysis was used to evaluate the relationship between continuous variables.

JMP version 8.02 (Cary, NC; USA) and SAS version 9.2 (Cary, NC; USA) were used for the statistical analysis. Significance was assigned at p< 0.05.

## RESULTS

### DLL1 expression is epigenetically regulated in GC cell lines

**Figure 1:**
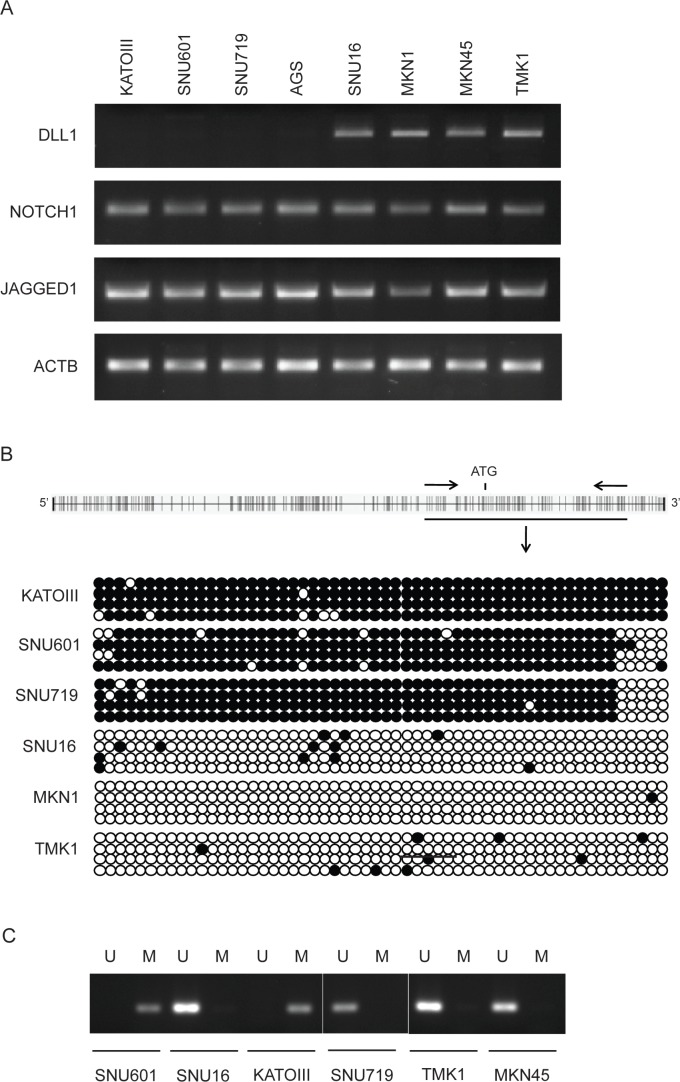
DLL1 expression and promoter methylation in GC cell lines (A) RT-PCR for DLL1, Notch1 and Jagged1. β-actin (ACTB) is used as housekeeping gene (B) Bisulfite sequencing: each dot represents a single CpG (black dot for methylated CpG, white dot for unmethylated). (C) Representative MSP (Reg 2, U=unmethylated; M=methylated).

In order to evaluate a possible role of the Notch system in gastric carcinogenesis, we screened a panel of 8 GC cell lines for the expression of Notch1 and its ligands DLL1 and Jagged1 at the RNA level by RT-PCR. We found that Notch1 and Jagged1 were expressed in the entire panel, while DLL1 varied quantitatively with the cell lines and was absent in KATOIII, SNU601, SNU719 and AGS (Figure 1A). Since the DLL1 promoter region is characterized by the abundant presence of CpG clusters, we hypothesized that an epigenetic mechanism could regulate DLL1 expression in GC cell lines. To assess whether the DLL1 promoter hypermethylation was critical for the suppression of DLL1 expression, the DLL1 promoter, 5’UTR and 1^st^ exon, spanning from −158 to +343 were analyzed by sodium Bisulfite Sequencing. Our data showed a strict correlation between DLL1 expression and methylation status. A dense methylation was found in non-expressing cell lines (KATOIII, SNU601, SNU719) while scattered methylated-CpGs were found in expressing cells (TMK1, SNU16, MKN1) (Figure 1B). These results allowed us to design a MSP on two representative regions (Reg1 and Reg2) (Figure 1C).

### DLL1 activates Notch1 signaling in GC cell lines

**Figure 2:**
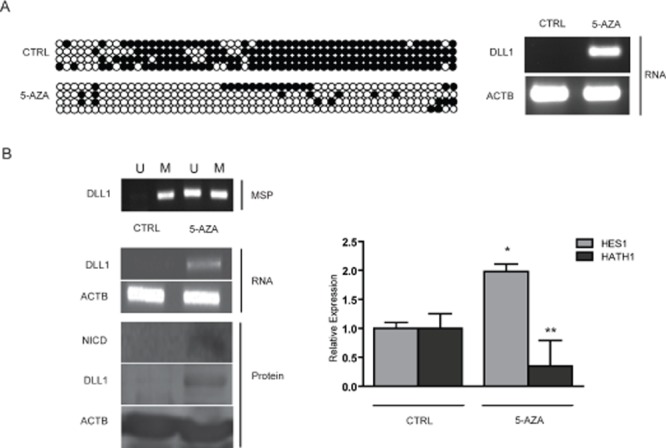
Treatment with 5-aza-2’deoxycitidine on GC cells (A) RT-PCR for DLL1 and bisulfite sequencing on AGS pre and post 5AZA treatment. (B) MSP for DLL1, RT-PCR for DLL1, Western Blotting for DLL1 and NICD and relative expression of HES1 and HATH1 evaluated with qPCR on SNU719 pre and post 5-aza-2’deoxycitidine treatment. (* p=0.0018; ** p<0.0001). 5AZA: 5-aza-2'deoxycitidine; Beta-actin (ACTB) is used as housekeeping gene.

**Figure 3:**
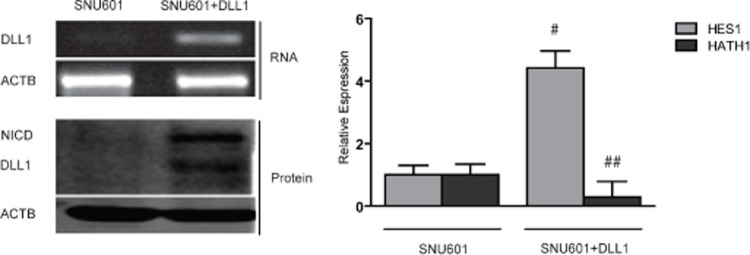
Over-expression of DLL1 in SNU601 RT-PCR for DLL1, Western blotting for DLL1 and NICD and relative expression of HES1 and HATH1 evaluated with qPCR (# p< 0.03; ## p<0.007). 'eta-actin (ACTB) is used as housekeeping gene.

Since we found a strict correlation between absence of DLL1 expression and promoter hypermethylation, we evaluated whether DLL1 level could be increased in non expressing cell lines by treatment with the demethylating agent 5-aza-2’deoxycitidine (5-aza-2’dC). To explore this hypothesis, Bisulfite Sequencing of DLL1 promoter pre and post 5-aza-2’dC treatment was performed on the AGS cell line together with analysis of the DLL1 transcript by RT-PCR (Figure 2A). We found that treatment with 5-aza-2’dC on AGS caused DLL1 promoter demethylation and, concordantly, an increased expression of DLL1 RNA. In order to confirm these results, the treatment with 5-aza-2’dC was performed also on SNU719 which also showed increased expression of DLL1 RNA and protein (Figure 2B). Moreover, we tested the effect of DLL1 on Notch1 signaling pathway activation. Interestingly, DLL1 expression caused an increase in cleaved Notch1 intracellular domain (NICD) protein together with a significant increase in HES1 (p=0.0018) and a decrease in HATH1 (p<0.001) mRNAs (Figure 2B). In order to confirm that DLL1 would trigger Notch1 activation, we transiently transfected SNU601 with the full DLL1 coding sequence. As shown in Figure 3, DLL1 ectopic expression resulted in increased expression of NICD with a consequent significant increase of HES1 (p=0.03) and decrease of HATH1 mRNAs (p < 0.007).

### DLL1 methylation in clinical samples

**Figure 4:**
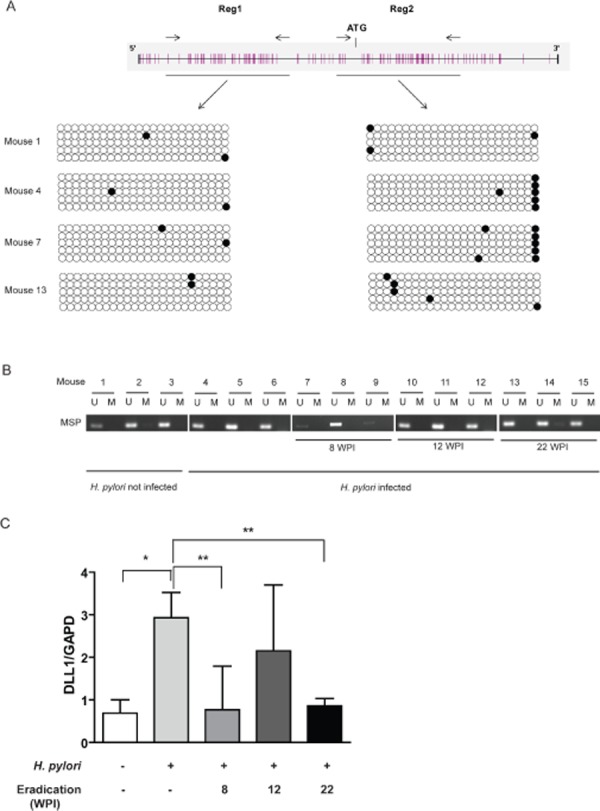
Methylation and expression analyses in GC patients (A) Distribution of GC samples according methylation status and Lauren’s classification. (B) Correlation between DLL1 and HES1 mRNAs in GC patients. (C) Association between HATH1 expression and diffuse and mixed histology (*p<0.0009;**p<0.005)

We examined the methylation status of the DLL1 promoter by MSP in GC patients (n=52) and healthy controls (n=21). In the GC group, DNA was selectively extracted from the cancerous area of formalin fixed paraffin embedded tissues. Our data showed that 20 out of 52 primary tumors (39%) exhibited aberrant DLL1 promoter hypermethylation in at least one region but not in any of the samples from healthy controls. We then correlated the molecular analysis with the pathological characterization, according to Lauren’s classification, and we found a significant association between the diffuse histotype and the methylation status in the Reg2 of DLL1 promoter (Figure 4A); it was hypermethylated in 11/24 (46%) of the diffuse type of GC samples versus 1/24 (4%) of the intestinal type (p<0.0009). Interestingly, hypermethylation in both regions (Reg1 and Reg2) was a unique feature of the diffuse type. No methylation was found in any mixed type GC for Reg2 (Figure 4A).

### DLL1 expression in clinical samples

In order to assess whether the methylation status of the DLL1 promoter could affect the expression of DLL1 and downstream targets, we performed quantitative PCR (qPCR) on RNA extracted from fresh GC patients (n=43) and healthy subjects (n=21) for DLL1, HES1 and HATH1.

Our data demonstrated a significant positive correlation between DLL1 and HES1 expression levels (r=0.6) (Figure 4B), being significantly stronger in the intestinal (p<0.008) or mixed type (p<0.007). A trend towards a negative correlation was found between HES1 and HATH1 in the diffuse and mixed types. However, when we correlated the expression of the individual genes with the pathological characterization of the tumor type, HATH1 expression was specifically associated with diffuse and mixed histology (p<0.005) (Figure 4C).

### DLL1 expression is not epigenetically regulated in INS-GAS mice

**Figure 5:**
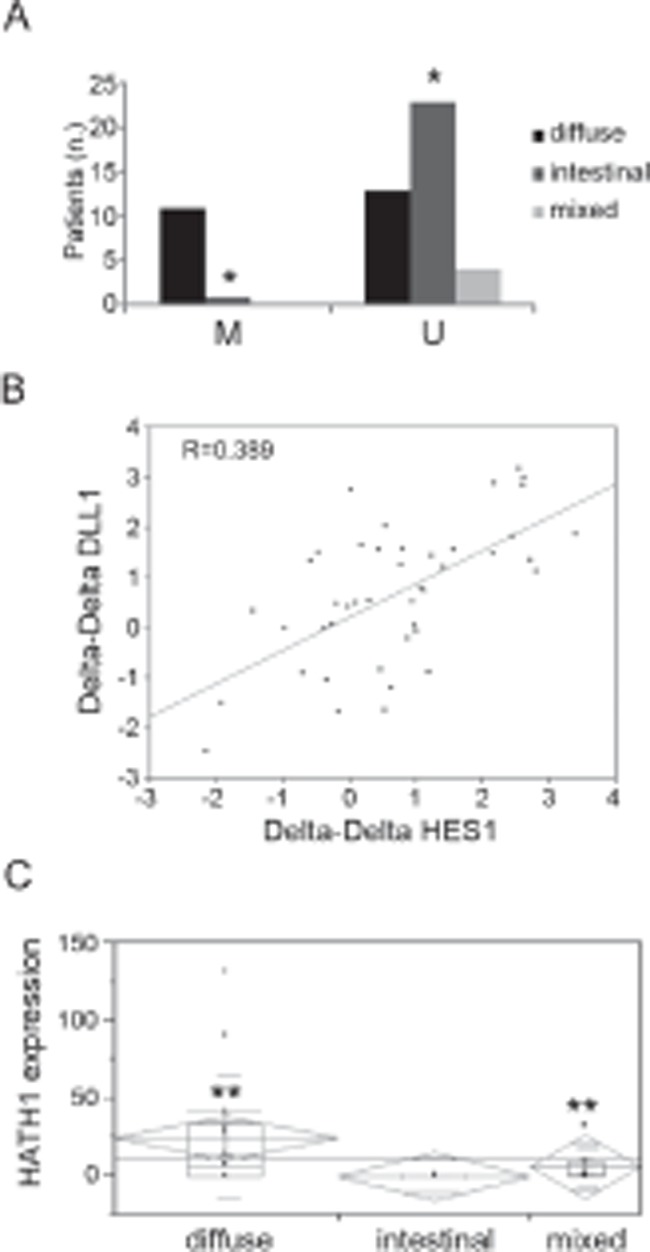
INS-GAS mice (A) Bisulfite sequencing performed in two regions of DLL1 promoter on DNA of gastric tissues from four mice. Each dot represents a single CpG (black dot for methylated CpG, white dot for unmethylated). (B) MSP for DLL1 on DNA of all the 15 mice. (C) Mean relative expression of DLL1 mRNA in each group of mice evaluated with RT-PCR. GAPD is used as housekeeping gene. WPI= Weeks Post Infection. *p=0.025; **p<0.05.

We investigated DLL1 promoter hypermethylation and mRNA expression on 12 gastric tissues obtained from *H. pylori*-infected and 3 gastric tissues from uninfected transgenic control INS-GAS mice treated at prescribed intervals with antibiotic eradication therapy [19]. The *H. pylori*-infected mice displayed several mucosal abnormalities with progression of the gastritis to intestinal-type gastric cancer. DLL1 promoter hypermethylation was evaluated by Bisulfite Sequencing (Figure 5A) and MSP (Figure 5B). We found no DLL1 promoter hypermethylation and, concordantly, DLL1 was expressed in each tissue sample analyzed (Figure 5C). We found a significant increase in DLL1 expression in *H. pylori*-infected mice compared to uninfected (p=0.0253) control mice, and a decrease in DLL1 expression in gastric tissues from mice treated with the antibiotic eradication therapy at 8 and 22 weeks post infection (p<0.05).

## DISCUSSION

Notch signaling is a key pathway in the self renewal of stem cells, cell fate determination and differentiation during embryonic and postnatal development and adult cell homeostasis [4, 13, 20]. However, it is known that Notch can function as an oncogene in several tumors [7], such as T-cell acute lymphoblastic leukemia, breast, ovarian and non-small cell lung cancer, with only one exception, in the keratinocytes of the epidermis, where Notch acts as a tumor suppressor [7, 21-22].

In the stomach, Notch signaling activation is involved in the developmental phases, controlling the commitment of the glandular differentiation and repressing the gastric mucin expression. In contrast, its inhibition is required for entero-endocrine cell fate determination [6, 12, 14, 16]. Recently, Notch signaling activation in lineage-committed stomach epithelial cells has been shown to trigger de-differentiation into stem cells, eventually enhancing proliferation and inducing adenomas with focal Wnt activation [13]. Moreover, Notch1 activation has been associated with gastric cancer progression, at least in part through cyclooxygenase-2 [23]. However, the specific role of the Notch1 ligand DLL1 in gastric carcinogenesis is still unclear.

We studied the Notch1 cascade in a broad panel of GC cell lines evaluating the expression of Notch1 and its ligands DLL1 and Jagged1 at the RNA level. We found no differences in the expression of Notch1 and Jagged1 among the entire panel while DLL1 was selectively expressed in SNU16, MKN1, TMK1 and MKN45. In contrast, KATOIII, SNU601, AGS and SNU719 showed a ‘reciprocal phenotype’ where DLL1 was absent due to promoter hypermethylation. The epigenetic regulation of DLL1 in these cell lines was further confirmed by treatment with 5-aza-2’dC in AGS and SNU719 that resulted in up-regulation of DLL1. Importantly, the increase in DLL1 expression after 5-aza-2’dC treatment resulted in activation of the Notch1 cascade with changes in the downstream targets HES1 and HATH1. In agreement with these data, the overexpression of DLL1 in SNU601 confirmed that DLL1 controls Notch1 activation.

According to Lauren’s histological classification, GC are divided into intestinal and diffuse types, associated with differences in etiology, epidemiology, genetic alterations, clinical behavior and response to therapy [24-26]. Gastric cancer of the intestinal type progresses as a multistep process that is initiated by inflammation, mainly due to *H. pylori* infection, followed by atrophy, intestinal metaplasia, dysplasia and cancer [27-28]. In contrast, the diffuse cancer type lacks precancerous phases and genetic changes underlying its initiation and progression still remain unclear, although promoter hypermethylation of several genes has been described as a frequent feature in the diffuse histotype [25, 29-30].

In the present study, the analysis of the Notch1 cascade was extended to clinical samples, evaluating the expression of DLL1, HES1 and HATH1 mRNA. We found that the positive correlation between DLL1 and HES1 expression levels was significantly stronger in the intestinal or mixed type, and as well, a trend toward a negative correlation between HES1 and HATH1 was found in the diffuse and mixed types. Interestingly, when matching HATH1 expression with the histological characterization, we found an association between a higher level of HATH1 and the mixed or diffuse histotype. Although HATH1 is not expressed in the normal stomach [11], a higher expression of HATH1 in gastric cancer compared to normal mucosa has been reported [31]. This finding confirmed previous reports indicating higher level of HATH1 expression in mucinous and signet ring colorectal cancer samples [32]. Furthermore, our data suggest that the differences in DLL1 expression are at least partially controlled by epigenetic changes in the DLL1 promoter. DLL1 hypermethylation was found as a specific feature of diffuse type gastric cancer and characterized almost 50% of this histotype. Interestingly, although HATH1 is associated with the diffuse type of carcinogenesis, our data showed hypermethylation of DLL1 promoter only in half of the cases. Our data do not exclude that other molecular pathways can contribute to HATH1 regulation and histological differentiation in the remaining cases. In fact, in the stomach, there is a complex network of cross-regulatory interactions among Notch and several other pathways, including Wnt, BMP and Sonic Hedgehog [33-34]. It has been demonstrated that HATH1 can be degraded by Wnt signaling [35-36] and HES1 expression can be controlled by Sonic Hedgehog [37]. On these basis, we can also hypothesize that the interaction among these pathways can contribute to HATH1 regulation and to diffuse type differentiation independently from DLL1 expression.

A further confirmation that the DLL1-Notch1 axis influences histological differentiation in GC arises from an extensive analysis of DLL1 promoter methylation and mRNA expression in *H. pylori* infected INS-GAS mice treated at prescribed intervals with antibiotic eradication therapy. Indeed, it’s known that *H. pylori* infected INS-GAS mice are a model of the intestinal type of gastric carcinogenesis [38]. In this model we determined that the DLL1 promoter is unmethylated and that DLL1 is expressed in all the mice analyzed. Interestingly, *H. pylori* infection causes an up-regulation of DLL1 and concordantly the antibiotic eradication therapy causes a decrease in DLL1 mRNA levels. This is consistent with published findings that DLL1 controls Notch 1 activity in mouse intestinal tissue [15] and Notch 1 activation in patients with intestinal-type of gastric cancer [39].

Among the GC histotypes, the diffuse form is associated with higher frequency of peritoneal dissemination, metastasis and mortality [25, 40]. In the present study we demonstrated that a subset of diffuse type GC lacks a functional Notch system, consistent with recent results reported by others [39]. These observations indicate that in certain kind of cancers Notch inhibitory agents, such as gamma-secretase inhibitors, might be useless. This data argues that by performing accurate molecular characterizations, groups of patients and/or subtype of cancers who can benefit from Notch inhibition can be accurately identified [41-43].

In conclusion, we demonstrated that DLL1 is epigenetically regulated in GC cell lines and DLL1 expression activates Notch1 signaling. In contrast, in the diffuse type of GC, DLL1 epigenetic silencing represses the activation of Notch and is associated with high level of HATH1, which is a specific feature of diffuse of mixed type of gastric cancer. Our results provide evidence that Notch1 activation in GC is controlled by the epigenetic silencing of the ligand DLL1, and that Notch1 inhibition is associated with the diffuse type of gastric cancer.
